# Supplementary material for: A haplotype-phased genome of wheat stripe rust pathogen Puccinia striiformis f. sp. tritici, race PST-130 from the Western USA
Source: PLoS One. 2020 Nov 11;15(11):e0238611. doi: 10.1371/journal.pone.0238611 (PMC7657539; doi:10.1371/journal.pone.0238611)
Supplement: Attachment — Submitted filename: Response to Reviewers (1).pdf [file pone.0238611.s001.pdf]

Answer to Reviewers and Editor

Editor recommendation

If applicable, we recommend that you deposit your laboratory protocols in protocols.io to enhance the reproducibility of your results. Protocols.io assigns your protocol its own identifier (DOI) so that it can be cited independently in the future. For instructions see: http://journals.plos.org/plosone/s/submission-guidelines#loc-laboratory-protocols

Answer: We added the doi link to protocols.io and the corresponding reference, and we rephrased the old sentence to: "In order to extract high quality high molecular weight DNA from the stored spores, we followed the protocol previously published in protocols.io (DOI: doi:10.17504/protocols.io.ewtbfen [22])."

[22] Schwessinger B, Rathjen JP. Extraction of high molecular weight DNA from fungal rust spores for long read sequencing. Methods Mol Biol. 2017;1659:49-57. doi: 10.1007/978-1-4939-7249-4_5. PMID: 28856640.

---

5. Review Comments to the Author

Reviewer #1

1. Materials and Methods contains only about fungal growth and DNA extraction. The methods utilized in the bioinformatics analyses are merged with the results (eg. lines 148 - 153, 156 - 167, 174 - 184, 187 - 193, 281 - 284, 322 - 333, 271 - 380, etc.) need to be placed in the Materials and methods section; and the results section should present only results.

Answer: As requested by the reviewer, we generated a new section designated "Bioinformatics methods" compiling all the bioinformatics methods into Material and Methods. We excluded those paragraphs from the results sections.

2. Lines 534 - 550: Be careful not to be too speculative about the hypothesis of one pre-2000 and one post-2000 haploid genome in Pst-130 as the supporting references presented may not strongly support the hypothesis. For example, drastic changes in virulence structure post-2000 may be associated with changes in the differential lines used to designate Pst races (Wan and Chen, 2014). In addition, Liu et al. (2017) did not discuss/confirm absence of sexual recombination in Pst as presented in the manuscript. The appropriate literature here is Wang et al. (2012); not withstanding Jin et al. (2010)

Answer: We agree with Reviewer #1 that the presence of a pre-2000 and a post-2000 haploid genome in PST130 is just a hypothesis and we modified the sentence in the Discussion to present it as a hypothesis rather than a suggestion: "Based on the different comparisons presented in Fig 4, we hypothesize that PST-130 (and maybe other post-2000 races of the Western USA) may have some genomic regions in common with the "Warrior" races from Europe, at least in one of their haploid genomes [19]."

We also agree with Reviewer #1 that the change of rust differentials in the determination of *Pst* races nomenclature introduces an additional layer of complexity. However, our comment about the number of post-2000 races is based on the original set of differential lines. Using the old PST nomenclature Wan and Chen (2014) reported an increase from race PST78 in the year 2000 to race PST147 in 2010 (70 new races!). We agree with Reviewer #1 that we were not using the correct citations for the number of race and for the absence of sexual recombination. Therefore, we rephrased the sentence to: "Between the initial PST-78 race in 2000 to PST-147 in 2010 (using the old *Pst* nomenclature) [45], 70 new *Pst* races were identified in spite of the absence of confirmed reports of sexual recombination in the region [46]. Somatic hybridization followed by nuclei exchanges between the new races introduced in 2000 and the local *Pst* races (without requiring sexual recombination) is a potential explanation for this phenomenon [8-11] that will require further validation."

References [45] and [46] are now:

45. Wan AM, Chen XM. Virulence characterization of *Puccinia striiformis* f. sp. *tritici* using a new set of *Yr* single-gene line differentials in the United States in 2010. Plant Dis. 2014;98:1534-42.

46. Wang MN, Chen XM. Barberry does not function as an alternate host for *Puccinia striiformis* f. sp *tritici* in the U. S. Pacific Northwest due to teliospore degradation and Barberry phenology. Plant Dis. 2015;99(11):1500-6.

The new reference of Wang et al. 2015 specifically discusses sexual recombination of stripe rust and the limited role of Barberry in *Pst* sexual cycle in North America.

Reviewer #2

The submitted manuscript (PONE-D-20-25928) by Vasquez-Gross et al. on a haplotype-phased genome of wheat stripe rust pathogen Puccinia striiformis f. sp. tritici (Pst) race PST-130 from the western USA is a well-designed study. The studies were successful in generating an improved genome assembly of a virulent race PST-130. The strength of the studies is that the authors combined PacBio technology (especially long PacBio reads) with Illumina sequencing (for error correction) and the Falcon-Unzip assembler which significantly improved the genome assembly of PST-130 and expanded the number of predicted genes. As concluded by the authors, I agree that the sequencing of more Pst phased genomes and future improvements on the phasing of complete haploid genomes will provide a much clearer picture of relative importance of sexual recombination and swapping of complete nuclei in the evolution of new virulences in Pst. The findings are of high topical interest and will be useful for many researchers working in area of pathogen genome assembly. The MS is written clearly, and the experiments are well planned, conducted, analysed thoroughly and interpreted. The results are properly discussed and well concluded.

The figures submitted were not of high resolution (at least in my download copy – they appear scanned version) and definitely needs improvement if the MS get published.

Answer; we have images of higher resolution but when we run it to the PACE web site requested by PLOS, they were transformed into lower resolution to comply with PLOS requirements.

I recommend the MS for publication with some minor comments:
1) In the abstract, authors mention very virulent race. What VERY virulent means? Also, in line 87, it is mentioned 'new more virulent'. What is more?

Answer: We eliminated the world VERY from the abstract. We rephrased as "… a post-2000 virulent race from the Western USA designated as PST-130"

We also replaced line 87 and eliminated the sentence "new more virulent". The new sentence describes more precisely the virulence profile of the Warrior races and adds a new reference [19]

PST-DK0911 was collected in Denmark in 2011 and belongs to the *Pst* race group known as "Warrior", which combine virulence to *Pst* resistance genes *Yr1*, *Yr2*, *Yr3*, *Yr4*, *Yr6*, *Yr*7, *Yr*9, *Yr17*, *Yr25*, *Yr32*, and *YrSP* [19]. This group is different from previous European races and similar to isolates from the near-Himalayan region, where they likely originated from sexually recombining populations [19].

[19] = Hovmøller MS, Walter S, Bayles RA, Hubbard A, Flath K, Sommerfeldt N, et al. Replacement of the European wheat yellow rust population by new races from the centre of diversity in the near-Himalayan region. Plant Pathol. 2016;65(3):402-11.

2) Line 105 – '21 days old plants of tetraploid wheat Kronos' will read better.

Answer: Edited as requested

3) Line 245 – Put 134 in bracket or revise the sentence.

Answer: We rephrased as: "Eighty nine percent of these contigs (134) are longer than 50 kb."

4) Line 529-530 - PST-130 may have some genomic regions in common with the "Warrior" races (Put a reference for Warrior race)

Answer: Added reference [19]

5) Table 4 – Put '% aligned' instead '% of aligned'

Answer: Edited as requested
